# “At Least until the Second Wave Comes…”: A Twitter Analysis of the NHS and COVID-19 between March and June 2020

**DOI:** 10.3390/ijerph18083943

**Published:** 2021-04-09

**Authors:** Kathy McKay, Sarah Wayland, David Ferguson, Jane Petty, Eilis Kennedy

**Affiliations:** 1 Public Health, Policy and Systems, Institute of Population Health, University of Liverpool, Liverpool L69 3GL, UK; 2 Tavistock and Portman NHS Foundation Trust, London NW3 5BA, UK; jpetty@Tavi-Port.nhs.uk (J.P.); ekennedy@Tavi-Port.nhs.uk (E.K.); 3 School of Health, University of New England, Armidale 2350, Australia; swaylan2@une.edu.au; 4 Formerly, Faculty of Transdisciplinary Innovation, University of Technology Sydney, Sydney 2007, Australia; dave.x.ferguson@gmail.com; 5 Research Department of Clinical, Educational & Health Psychology, University of College London, London WC1E 6BT, UK

**Keywords:** COVID-19, NHS, twitter, content analysis, moral injury

## Abstract

In the UK, tweets around COVID-19 and health care have primarily focused on the NHS. Recent research has identified that the psychological well-being of NHS staff has been adversely impacted as a result of the COVID-19 pandemic. The aim of this study was to investigate narratives relating to the NHS and COVID-19 during the first lockdown (26 March–4 July 2020). A total of 123,880 tweets were collated and downloaded bound to the time period of the first lockdown in order to analyse the real-time discourse around COVID-19 and the NHS. Content analysis was undertaken and tweets were coded to positive and negative sentiments. Five main themes were identified: (1) the dichotomies of ‘clap for carers’; (2) problems with PPE and testing; (3) peaks of anger; (4) issues around hero worship; and (5) hints of a normality. Further research exploring and documenting social media narratives around COVID-19 and the NHS, in this and subsequent lockdowns, should help in tailoring suitable support for staff in the future and acknowledging the profound impact that the pandemic has had.

## 1. Introduction

A recent editorial in the Lancet Planetary Health called the current times the “pandemic era” where “despite repeated warnings about the very real risk of occurrence from infectious disease experts, it felt remote and distant, not something for most people to worry about day to day” [1] (e1). While COVID-19 is a global pandemic, the ways in which governments and health care services around the world have dealt with the prevention of transmission of COVID-19, the provision of care for patients and the protection of health care staff have differed significantly. Consequently, the COVID-19 pandemic and government and health care response are subjects that people consistently worry about during their everyday lives, and which have also impacted their interactions with both traditional and social media; see [2,3].

The first COVID-19 cases in the UK were reported on 31 January 2020, with the first local death confirmed on 5 March 2020. The first lockdown to be enacted in the UK started on 26 March 2020 and continued until 4 July 2020. There have been two lockdowns since: the second from 5 November 2020 to 2 December 2020, and a third announced on 4 January 2021, which at the time of writing was still on ongoing. During all three lockdowns, various guidelines have been put into place including travel restrictions, work/school from home mandates, and restrictions on meeting people from outside a household. At the time of writing (6 February 2021), the UK has just passed the peak of a third wave of COVID-19, with 112,000 deaths reported, and over 32,000 in January 2021 alone, considered to be the highest death toll in the world per capita from COVID-19, with an estimated one in 660 UK citizens dying from the virus to date [4,5,6].

The communication of health-related information in this context is clearly vital [7]. Traditional media ostensibly remains a key provider of information. In the UK, press conferences have been televised to both keep the public up to date with the rates of infection and death in their local areas and around the country, as well as to announce local and national lockdowns and the restrictions within them. Studies around the communication of health-related information during the SARS epidemic in Hong Kong in 2003 demonstrated the importance of clear messaging when dealing with a public’s fear of a previously unknown virus, as well as the way in which perceptions could and did change throughout the epidemic [8,9]. Further, Wallis and Nerlich [10] argued that the way in which UK newspapers framed SARS helped to create a language of a virus as a ‘killer’ and how people’s lives might be saved. However, as noted by Pearman and colleagues in January 2021 [11], worldwide news coverage of COVID-19 declined after late March 2020, including in the UK, despite the fact that reported infections and deaths continued to increase until recently.

While there may have been at times a decreasing presence in traditional media, COVID-19 is very much a consistent and vocal presence on social media. Further, social media offers the public a chance to engage with different issues around COVID-19 in an active way compared to a potentially more passive consumption of traditional media. For example, although traditional media did focus on the high death toll and extreme pressure on the health service from January 2021, this was proceeded by a persistent focus on social media in the preceding months. While other papers have examined twitter usage by world leaders [12], health professionals [3], or conspiracy theorists [13], the focus of this paper is to examine Twitter users’ perceptions of COVID-19 and the UK’s National Health Service (NHS). The NHS was established in 1948 in the aftermath of the second world war and is one of the most comprehensive public health care systems in the world, providing free care according to need to millions since its inception. While it may no longer be considered the ‘envy of the world’, it continues to symbolise the idealism of the 1942 Beveridge report, leading to the foundation of the modern welfare state in the UK [14]. As such, the NHS holds a particular position in the UK national psyche and has not infrequently been described as a ‘national religion’. Recent research has also shown that the well-being of NHS staff has suffered as a result of the continuing pressures of COVID-19 [15]. Further, the well-being of health care staff internationally has been of great concern as the pandemic continues [16,17,18]. This paper seeks to identify and investigate narratives around the NHS and COVID-19 from the first lockdown in order to better understand how people perceived the work being done within the NHS, the actions of others outside the NHS, and what this meant to them during the first lockdown in order to identify relevant learning, particularly as the UK is now in a third period of national lockdown.

## 2. Materials and Methods

Using the Twitter API software, DF collated and downloaded tweets bound to the time period of the first lockdown in order to analyse the real-time discourse around COVID-19 and the NHS. Tweets were extracted using the twitter API, R Studio software and the R package rtweet. Tweets were extracted daily and added to an R data file. This is in line with tweet collection strategies in previous research [19]. Hashtags were chosen by the research team based on relevance for the time period, as well as allowing for a broad range of tweets to be collected. The hashtags searched were #NHS, #SaveOurNHS, #COVID19UK, #COVID_19, #PPE, #ClapForOurNHS, #ClapforOurCarers, #BooForBoris, and #BooBoris. The hashtag #COVID19Ireland was also included but recovered a small number of tweets and never appeared in tweets by itself that were included in the analysis. The hashtag #COVID19 was only searched for when it was included in addition to any of the others. Altogether, 123,880 tweets were collected and included in the analysis. The information collected with each tweet included time and date it was sent, text, tweet ID, handle, link, number of favourites, number of retweets, mentions (of other twitter users), first search term, number of search hits, potential retweet, hashtags, and tweet month. While location data are rarely included in tweets, and so could not be used to geographically bind the tweets collated and downloads, the hashtags themselves tended to limit the tweets to the UK COVID-19 experiences.

In line with the process set out by Bengtsson [20], content analysis was undertaken by KM, who identified the concepts, categories and themes. KM examined not just the words of the tweets, but the relational aspects of them—whether a tweet identified a particular person/group or experience, or whether a tweet was grounded in the individual or was more community focused. Tweets were coded around positive and negative sentiments [21]. Positive sentiments used language that was encouraging, supportive, or praised particular people or groups. Negative sentiments used language that was derogatory, angry, or upset with particular people or groups. KM checked concepts and categories with EK and JP, with JP reviewing the data set. The themes then identified were checked for consensus with all the authors. Themes were unpacked alongside the way in which hashtags were engaged with over time, and the emotion they evidenced in not only the language used but who the tweet was directed towards. In this way, a narrative of emotion was uncovered: feelings of support and disappointment, hero worship and betrayal.

Following the data collection guidance set out by Pedersen and Lupton [22] and Fiesler and Proferes [23], no ethical approval was needed for data collection as all tweets collected were in the public domain, which excluded any closed or private accounts, and all tweets included were ‘spontaneously generated’ rather than elicited. In addition, while the tweets are dated, handles are not included with any tweets used within the findings to further assist in anonymisation.

## 3. Results

The analysis of the data included 123,880 tweets. In this section, we explore the timeline by which tweets are shared, and the ways in which they are engaged with. What was identified was that when lockdown one started, there was significant tweeting and engagement, particularly with the hashtags around #clapforcarers. However, by the week of 11 May, there were generally fewer tweets and lower levels of engagement. After this time, peaks of engagement tended to occur with controversies linked to government handling of the crisis. Table 1 shows the number of tweets per week by hashtag.

The majority of tweets were shared by individuals, rather than organisations. Of the top 20 tweeters (defined as those who tweeted the most using the relevant hashtags), five were organisations—four of which were directly health related. Of the 15 individual top tweeters, two worked within the NHS; the top tweeter included ‘Dr’ in their handle but it was uncertain whether this meant they also worked in the NHS or had professionally recognised qualifications.

Five themes were identified:

1. The dichotomies of ‘clap for carers’:
  - Positive perceptions, and
  - Negative perceptions.
2. Problems with personal protective equipment (PPE) and testing.
3. Peaks of anger:
  - Lockdown breaches and implications, and
  - Preventable (and endless) deaths.
4. Issues around hero worship.
5. Hints of a normality.

### 3.1. The Dichotomies of ‘Clap for Carers’

The ‘#clapforcarers’ tweets formed an organised narrative across social media each Thursday from 2 April 2020. Tweets started in the morning and continued throughout the day: a simple tweet saying when the clap was happening, with the person who tweeted stating their commitment to ‘join in’, and encouraging others to physically engage in clapping at the allocated time. In later weeks, some tweets would dedicate their clapping to a particular person or part of the NHS (particularly NHS staff who had died from COVID-19). Later tweets often referred back to the clapping of previous weeks, encouraging people to be louder or to remember to join in. Some tweets appeared to be coordinated, particularly from organisations. These included emergency services, football clubs, and even stereotypically British children’s programs such as The Wombles. All major and fringe political parties tweeted in support of the NHS too. As well as encouraging people to clap in support of the NHS, emergency services tweets also offered advice about how to clap safely. For example, on 9 April 2020, one police service tweeted:

In ten minutes, please support our wonderful @NHSuk staff and carers in the battle against #COVID19 by clapping from your property. Please make sure when you clap you socially distance #HeartForHerts #ClapForCarers #StayHomeSaveLives #ProtectTheNHS #WelwynHatfield

By the last weeks in May, the main tweets around clapping were only during Thursday rather than throughout the week. The last organised clap was on 28 May 2020 and the hashtags significantly decrease after this.

#### 3.1.1. Positive Perceptions

The vast majority of the tweets around the clapping hashtags were positive, where the language used was supportive, happy, and looking forward to it. Within the tweets, there seemed to be a sense of community and belonging that felt very important to the people tweeting when the world seemed completely different during the lockdown. Small villages could make just as much noise in support of the NHS as any city. There was substantial pride and gratitude towards the NHS, an institution that was framed as intrinsically British. Buildings, particularly those at the centre of cities and towns, turned on blue lights in solidarity during the lockdown. This appreciation was exemplified in a tweet towards the end of the first lockdown, after the clapping but when people had started to digest the extent of the devastation during the first lockdown:

For anyone who have ever criticised the #NHS-of course it’s not perfect, of course mistakes are made, but this is the alternative. Imagine surviving #COVID19 but being left not only with severe disabilities but with a $1 million bill as well. (25 June 2020)

A lot of emotion was expressed through a person’s clapping, particularly it seemed if they did not work in the NHS themselves. At times though, it did not always appear that the enthusiasm was well thought through if more than hyperbole:

I clapped so long and hard last night I hurt my wrists and had to go to A&E. That’s how much I the NHS. Beat that. #ClapForOurCarers #NHS (8 May 2020)

The clapping was appreciated by many within the NHS, especially as they struggled with the work:

“Every day there’s risks, there’s things that can get us down but the support it really keeps us going so thank you.” [name] says that despite the physical, mental and emotional pressures on healthcare staff the support from the public motivates them. #ClapForCarers (3 May 2020)

#### 3.1.2. Negative Perceptions

However, some tweets sought to challenge the performative nature of the clapping. There was a growing sense that the clapping may have been a positive symbol, but it should not be in place of tangible requirements such as PPE and adequate salaries. Feelings of betrayal and anger were expressed at the NHS, and the workers within, being let down by the government with the clapping used as a distraction. These arguments were initially linked to the hashtags around ‘#BooforBoris’ which started on 4 April 2020 (examined in a later section). These tweets may have been fewer in number than the positive tweets but they were equally emotional. People really struggled with wanting to show support, but to also ensure their support was meaningful:

Although well intentioned, I think some like #clapforcarers because of the nice ‘bringing people together’ feeling. It isn’t giving health professionals better conditions, PPE, or a pay rise. Clapping is fine, but let’s insist on a future where nurses don’t need food banks. (17 April 2020)

“I find the clapping quite sinister. It’s as if it’s okay not to care about the neglect of the NHS.” [name] says #ClapForCarers helps us feel better about frontline workers being “treated like shit”. (24 April 2020)

This is beyond a fucking joke. Meaningless gestures of ‘thanks’ when frontline workers are being so terribly failed by our government, day in and day out. Just STOP!!! Do your jobs!! PROTECT the people who are DYING saving us. #PPE NOW!! #Covid19UK #CriminalNegligence #NHS (28 April 2020).

I feel really uncomfortable, unbearably uncomfortable going out & clapping tomorrow night. I want to show my support but when our care workers are dying every day through lack of #PPE I’m torn about what to do? Any advice? #ClapForOurCarers #ClapForTheNHS #clapforkeyworkers (28 April 2020)

Clapping seemed especially problematic on 17 April 2020 when images were taken of a large crowd, many not wearing masks or social distancing, clapping on Westminster Bridge. Arguments were made about the purely performative nature of this clapping: all show but putting yourself at risk of catching or spreading the virus that the NHS was struggling to control:

Let’s all gather in large groups (ignoring social distancing) and clap for the NHS ... You can’t make this stupid shit up! #westminsterbridge #clapping #ClapForCarers

Police slammed for standing and watching as dozens breach social distancing rules during #ClapForCarers #COVID19 #StayAtHome MORE: [link]

Here, ‘#COVIDIOTS’ started to be linked to tweets about clapping, alongside later arguments against the government and austerity.

As the clapping time moved into May, arguments about the performative nature of clapping increased: the tangible vs. symbolic, who was clapping and who was not clapping, and who was too performative. One tweet on 21 May 2020 labelled the clapping a “guilt easing spectacle”.

The clapping hashtag was also used to raise awareness about the proposed NHS pay freeze on 12 May 2020, again arguing about the symbolic over the tangible:

They’re going to freeze the wages of the very keyworkers who are saving us? I would strike to stop this and I know a few million more who would too #ClapForOurCarers

Further, some people felt stressed about the continuing nature of a weekly clap. How would they be perceived by others if they were not seen to clap one week? There was a sense in these tweets that the only way a person could show support was to clap, so without clapping they were not being supportive:

I definitely have sunstroke. I don’t think I can make it out for the #ClapForOurCarers but I don’t want my neighbours to judge me ... (14 May 2020)

In addition, other tweets argued for the inclusion of all key workers in these demonstrations of support, not just those in the NHS. Anyone outside of the NHS was perceived to be forgotten. This argument became especially heated on 14 May 2020 when the safety of schools reopening for both teachers and students was being discussed:

Utterly crass to suggest teachers haven’t been as brave as #NHS. Do we need more teachers to die of #COVID19 to prove risk is same? Teachers have been stepping up & to suggest otherwise is irresponsible as a parent, this tweet is appalling It is teacher shaming & bullying them

### 3.2. Problems with PPE and Testing

The intersection of politics and health was particularly prominent in the tweets around PPE where NHS staff were seen as having been betrayed due to the government’s inability to secure adequate supplies of PPE to protect them in their work. Access to testing for the virus was another fraught issue, where NHS staff were dependent on the government to establish effective testing systems. Those who tweeted about the problems with PPE and testing tended to question the priorities of the government in ensuring everyone had access to quality health care and virus prevention. Engagement was linked to discontent prior to the first lockdown about who had access to testing earlier in the year, where celebrity and wealth was argued to provide more access than being in the NHS.

The shortage of PPE sparked emotional engagement (with higher numbers of likes and retweet) particularly around 10 and 11 April 2020 as people started responding to statements from the government:

Multiple interventional procedures in full #PPE is v hard work. Long sleeved waterproof plastic aprons like jogging in a bin bag. Face wrecked-spots like a teenager! Hot & no AC to minimise air circulation. #NHS #COVID19 #IR @RCRadiologists @BSIR_News (10 April 2020)

As an #NHS cancer consultant who is trying to deliver a cancer service, whilst also being on a #COVID19 ward rota, there’s no way that I’ll remain silent & allow this Government to get away with the lie that “herd immunity” (without a vaccine) wasn’t prior policy. #accountability (11 April 2020)

Been contacted a nurse who has suggested @MattHancock does her shift where he will be offered “whatever #PPE is left over from day shift” just like she gets when she turns up. Sort it Health Secretary! #nhs #COVID19 (11 April 2020)

There was such concern around the lack of PPE that, continuing into May 2020, people were tweeting about how they were making ‘home made’ PPE (masks particularly) to donate to the NHS. That ordinary people felt driven to such action was again linked to tweets arguing government incompetency for this having to be done.

### 3.3. Peaks of Anger

#### 3.3.1. Lockdown Breaches and Implications

Two types of lockdown breaches caused considerable peaks of anger: governmental and public. During the first lockdown, tweets around governmental breaches occurred predominantly on 24–25 May 2020, when it was revealed that Dominic Cummings (a non-elected advisor to the Prime Minister) had travelled out of London with his family when they were COVID-19 positive in direct violation of lockdown guidance. The Prime Minister Boris Johnson was called upon to sack Cummings, which he refused to do. On 24 May 2020, many angry tweets gained traction:

From a different perspective, I follow quite a few UK accounts. Every single one is talking about Cummings Every. Single. One That never happens If Mr Johnson thinks this is going away, he is very much mistaken #booforboris

Cummings should be heckled and hounded wherever he goes. He’s the architect of misery and suffering for so many people. He needs to be sacked right now. #SackDominicCummings #borisspeech #truthtwisters #booforboris

Boris has stabbed us all in the back. His protection of Dominic Cummings is an insult to all of us. So many people have made huge sacrifices. #BooForBoris #SackCummimgs

People were so angry that ‘Boo for Boris’ started trending on the same day, where people encouraged each other to boo loudly at 7:55 p.m. just before the Thursday 8 p.m. clap for carers. This was the most momentum tweets around clapping for carers had in while. While a lot of very simple tweets just said “#BooForBoris Tuesday 8 pm”, others tried to turn their anger into humour:

Boris: “are they booing me?” Cummings: “No, they’re saying Boooooo-ris boooooo-ris” #booforboris

I’m going to do it Tuesday and get my full PA out. And perhaps the keyboard and/or clarinet. Any suggestion for appropriate tunes I could play? #booforboris

Interestingly, one tweet asked for the language to be changed, pointing out the familiarity of using a first name to identify someone compared to their surname:

Can we #JeerForJohnson instead? Calling him ‘Boris’ is playing his game. He’s not our chum. #BOOFORBORIS

At a time when clapping was scheduled, and living with the virus was a new normal, Cummings’ breach highlighted that symbolism was not enough when tangible acts such as this were felt to be so abhorrent. The breach resulted in people discussing balancing the difficulties of abiding by the lockdown guidance with the responsibility of protecting the NHS. There was a sense that it jolted some people out of normalising all the deaths during lockdown:

I can tell you the instincts of this father. I’ve had the virus. We’ve taken care of our kids alone. We’ve lost family from it supposedly surrounded by a ‘protective ring’. We’ve mourned alone. We’ve stayed home and followed the guidelines. #Hypocrites #booforboris #CumminsSacked (24 May 2020)

That strange time where it takes one guy to break the lockdown rules and not thousand of deaths in care homes to get Brits so angry.#booforboris #Dominicummimgs (25 May 2020)

Peaks of anger around breaches of lockdown by the public also used protecting the NHS as a cultural touchstone. The ‘NHS’ was invoked in tweets to promote people following lockdown as a reason to do ‘the right thing’ when health care workers were risking their lives to help others. The anger around public breaches of lockdown became despairing over a potential spike after VE Day on 9 May 2020. This is when language around not complying (and those who argue oppression) really starts to solidify and gain traction:

All you shitbags that were dancing in the streets, getting pissed and not giving a fuck for social distancing don’t you fucking dare #ClapForOurCarers next Thursday, don’t you fucking dare, apologise for my language, but this is #CoronavirusPandemic not seasonal fucking flu.

This is the sacrifice some are making, while others shout about the ‘oppression’ of masks and social distancing. Weeks without holding his daughter or partner. So he can continue saving the lives of others. #COVID19 #SARSCoV2 #NHS

The next day (10 May 2020) is when the arguments around whether people who flaunt the guidelines and then get sick deserve to be treated by the NHS also gain traction:

Maybe the people who don’t want to stay home should agree, that if they get #Covid19, they won’t ask the #NHS to save THEIR lives.

Unpopular view but people who feel #COVID19 is either a conspiracy, or just the flu bro, and deliberately break the rules should not be allowed to use the #NHS. Sorry I know this sounds harsh but I am sick and tired of selfish #COVIDIOTS #StayHomeSaveLives

These emotions and arguments are repeated on 16 May 2020 after images of crowds in Hyde Park are published.

#### 3.3.2. Preventable (and Endless) Deaths

The anger around the increasing number of COVID-19 deaths continued throughout the first lockdown. At first, some media had framed the NHS caring for COVID-19 patients in overcrowded hospitals with wartime analogies. This had begun to wear thin by 28 April 2020:

Stop clapping us, stop using fallen heroes, war time analogies just to make @borisjohnson feel Churchillian. Most medics are scared to work. Politicians you have let us down, shame on you. #Panarama #COVID19 #NHS #R4today #bbcnews @piersmorgan @Number10press @sheryllmurray

From 18 April 2020, tweets that were critical of the government’s handling (separate to the NHS) tended to be more interacted with than tweets simply praising the NHS:

Over 100 #NHS staff have died from #COVID19. We have every right to speak out & question the Government and it’s advisors on their handling of the COVID19 pandemic. We need to know the full truth behind their response, in order to best understand how to manage the ongoing crisis (21 April 2020)

The rising number of deaths among NHS staff was highlighted not only as a way to demonstrate the risk that NHS workers face just by performing their job, but that also many of these workers were immigrants. This became a powerful argument when the accessibility of work visas was questioned in parliament on 18 May 2020.

Further, it was important that deaths of NHS staff were not normalised as a casualty of ‘war’:

“It is not normal for people to die in the course of their work as a doctor, nurse or other healthworker or social care worker... each and every death should be regarded as a ‘never event’ in NHS language” #Covid19UK #NHS (2 May 2020)

In line with this, NHS workers shared the emotional impact that working with COVID-19 patients had on their well-being. This was far different to what they normally experienced and the disconnect at times between what they saw and the breaches of lockdown was upsetting:

Unable to sleep (again!) due to #COVID19 stress. Never suffered Insomnia before! Feel scared for my family & team. Mental health impact on doctors & frontline #NHS staff is very real. Hope people feel more able to share. We need kindness & protection too! @TheBMA #OurNHSPeople (12 April 2020)

The in car, pre work pep talk-you can do this [name]-and try not to cry all the way home afterwards this time #NHS #COVID19 #today #ambulance (16 April 2020)

Coming to the end of my 3 night shift. Emotional and tired. All patients asked me for was my hand. Images I saw yesterday of street parties upset me. Please #StayHomeSaveLives #helpless #covid19 #NHS #Doctor @Imperialpeople @acutemedicine @RCPLondon @piersmorgan–at Hammersmith Hospital (9 May 2020)

A minute of silence for NHS workers who had died was announced on 28 April 2020:

1 Minute silence at @BartsHospital for our NHS family lost to the Pandemic. #StayHome #COVID19 #NHSheroes #nhs–at St Bartholomew’s Hospital

However, there was not as much engagement with the tweets about this at all (few had more than 50 likes), which suggests a discomfort with the number of deaths compared to the clapping which was framed more positively.

On 4 May 2020, new bereavement guidance was issued:

New bereavement guidance has been produced by @NHSEngland to support #NHS staff following the loss of a colleague, friend or family member during #COVID19. Take a look at the resources

This acknowledged that grief and bereavement were not only significant issues in the present but will also continue long after the pandemic is ‘over’.

However, there were also updates on patients who survived, even when it was not expected. These tweets offered hope amidst the anger and despair:

Today we discharged our 302 covid + patient home, one month and a day since our first confirmed covid + patient. Hurrah! #NHS #COVID19 (13 April 2020)

### 3.4. Issues around Hero Worship

Connected to the symbolism of clapping was the narrative around the construction of NHS workers as ‘heroes’. In line with a wartime analogy, these heroes willingly put their lives at risk for the greater good. However, this symbolism was also problematised—what tangible benefit did it bring to the NHS in their quest to prevent people from dying during the pandemic?

Unpopular opinion: emotional hero-worship of the #NHS is not healthy. It will not secure its long term security. The wave of emotion will make it easier not harder for #NHS to be swept aside along with painful memories of #COVID19, when concern turns to economy (and austerity) (16 April 2020)

A hero worship narrative was also constructed, and problematised, around Captain Tom Moore. Moore was a former British army officer, who, in the lead up to his 100 birthday, decided to walk 100 laps of his garden to raise money for the NHS. There was overwhelming support for ‘Captain Tom’ and significant media coverage led to his raising over £32 million for the NHS:

At 99 (soon to be 100) @captaintommoore is an absolute legend! He’s walking 100 garden lengths before his birthday for our #Covid19 Urgent Appeal. Captain Moore, we’re with you every step of the way. #NHSThankYou #NHS (11 April 2020)

However, as proud as people were of this achievement, the question was raised as to why this had to be done. Could the government not fund the NHS properly? This argument was raised again when Captain became Sir Tom and he was knighted on 19 May 2020:

Predictable distraction. When did #NHS become a charity? Where is the money going? Why did Tom have to do this in the first place? Why don’t we remove profiteering parasites from #NHS rather than inviting more in under cover of #Covid19UK?

### 3.5. Hints of Normality

By the end of June 2020, a few NHS staff tweeted about their hospitals almost feeling like they were back to normal, although the staff themselves did not feel ‘back to normal’. However, this was heavily underlined by the knowledge that the second wave of the virus would arrive soon enough. This was a pause, not the end:

Very little #covid_19 in the hospital now and things returning to a sort of normality, but everyone just seems exhausted. I think most #NHS workers could do with a really good holiday about now (at least until the Second Wave comes ...) (21 June 2020)

In comparison, a tweet one week later highlighted that ‘normal’ now seemed to mean not in need of support. Tying into the arguments around the performative nature of the clapping, one health care worker questioned how supportive people really were when they could be so rude as patients:

Work was manic today. People expecting to just walk in. Patients being rude, swearing, demanding and obnoxious. I bet just a fortnight ago they were clapping on a Thursday night... Some memories are short lived. #NHS #COVID19 #NHSheroes (29 June 2020)

## 4. Discussion

The aim of this paper was to examine Twitter users’ perceptions of the NHS and their role during the COVID-19 pandemic, particularly focused on the time during the first lockdown in 2020. Analysing the tweets shared in real time during the first lockdown while living through the third lockdown has shaped the analytical approach in a way not always possible in research, where three of the authors have been based in an NHS Trust in London during the pandemic. The tweets analysed in this paper demonstrate how people within one country, using a specific form of social media, experienced a global crisis, and how an institution such as the NHS could provide a point of connection for many during such uncertainty. With accessible real-time data from social media available, there is an opportunity to understand what worked within a situation, and where more support or clearer communication may have been needed, so as to ensure the same mistakes are not continually made. More thought needs to be given as to how researchers can study something that they themselves are experiencing at the same time—a living experience, more than a lived experience. Fox and Wayland [24] examine how strong reflective practice can assist researchers in carrying out this work. While content analysis does not always allow the space for reflection, the authors have continually talked through how the themes reflect (or not) their own experiences and how these ideas have shaped their current experiences.

### 4.1. The Role of Social Media in Offering Voice

While the ways in which social media can give voice to the wider community about significant public health issues has started to be explored [25,26], this is the first time social media has been utilised during a major global pandemic. There was a real sense of shared uncertainty as people not only sought to process their experiences—of unexpected and life-changing illness, loss, and grief—but also parse the information being shared in a time where incorrect information could literally be life-threatening. The tweets demonstrate the passion of people’s emotions during the first lockdown, and how these changed over the months, particularly in terms of how people reacted to the increasing death toll and the (at times avoidable) risks that NHS and other essential workers faced, as well as the community in general when lockdown guidelines were broken. There was a sense of COVID-19 and lockdown fatigue after the first month, where the number of tweets decreased and there was slightly less engagement. However, the experience of lockdown, where life becomes more limited, may have also impacted on people’s tweeting habits. People may simply have not wanted to tweet about the same experiences, even if it was all they had. This analysis found that strong emotion was needed to reinvigorate engagement, particularly anger.

### 4.2. Social Media as a Space for Meaningful Conversation?

Tweets by themselves, by their very nature, can often lack nuance, particularly with complex issues. Analysed together though, they demonstrate not only the ‘loudest’ voices but also the process of meaning making while living through traumatic and stressful events. With dirty text, Tamas [27] explores how people become able to move through the mess of trauma to make sense of it, that stories are often non-linear and people can become stuck in narratives invisible to others. The tweets analysed in this paper demonstrate how people were ‘digesting’ the trauma of COVID-19 during the first wave, with little sense that there would be a second wave, let alone a third. These tweets are the public face of real-time meaning making. They are the precursor to the articles written by health care professionals later on in the pandemic about the fear, exhaustion, and grief of trying to save people’s lives [28,29].

### 4.3. Performative Acts as a Response to the Pandemic

The analysis highlighted how important the NHS is within the UK in both tangible and symbolic ways. Here, the clap for carers hashtag demonstrated the tangible—those working in the NHS frontline were literally trying to save people’s lives—and the symbolic—people clapped because they felt unable to help in any other way during a time that felt out of control for many. Indeed, it is the symbolism that is striking, especially at the beginning of the first lockdown as the numbers of cases and deaths increased. There are people who appreciate the clapping, and they are a mix of NHS staff and the general population, who find a sense of community and value in it, where it is positively received and perceived. However, the problematisation of the symbolism resonates all the more strongly from the perspective of the third lockdown, where cases and deaths have continued to rise at a higher rate than the first lockdown. The narratives of who is dying, and whether these deaths could have been prevented and the arguments around living wages for key workers pointed to a powerful disconnect between the tangible and the symbolic, and one that continues. The tweets from those within the NHS speak of the trauma that they experienced—related to a lack of PPE, and people dying in large numbers—and a trauma that is constantly still rumbling in the background. Now staff burnout has arguably replaced PPE as the prominent concern linked to the loss of life and ‘moral injury’ experienced by staff as NHS resources are stretched to the point where they are unable to ensure optimal patient care. There was clear agreement among the NHS staff who tweeted with these hashtags that wartime and battle analogies, especially when connected to hero worship of the ‘sacrifice’ made by them, were not helpful during a global pandemic. There is a larger conversation to be had about how appreciation translates into value, and when something well-meaning cannot offer the support that NHS workers actually need. This has led to the COVID-19 Bereaved Families for Justice group calling for a public enquiry with a rapid review phase so that lessons from the first stages of the pandemic can be applied going forward [30]. Further, the tweets which highlighted the disappointment, anger, and grief felt by NHS staff as a result of their experiences during the pandemic, particularly around the lack of PPE, and members of the public by proxy, demonstrate the importance of further research around how they may be impacted by this both now and in the future [31]. We not only need to understand how to best support NHS staff during the pandemic, but in its aftermath as they continue to navigate the psychological repercussions.

The disconnect between the tangible and symbolic also leads to a powerful narrative around those who are perceived to be following the lockdown guidelines and those who are deemed to have broken them. There is a real sense here that while clapping for carers may only ever be symbolic, following lockdown guidelines is a tangible way to help the NHS. The fewer people who get sick from COVID-19 and need to be hospitalised, the less the strain on the health care system. Indeed, there is real anger directed towards those who are perceived to be hypocritical—someone who claps but then breaks lockdown guidance. This is most pointed during the Cummings episode in May 2020, where symbolism appeared to break down entirely.

### 4.4. Strengths and Limitations

There are several strengths and limitations of this paper. Twitter can capture the feelings of a specific moment or time very well across a wide range of people—it provides data at a very grass roots level. People might tweet a stream of consciousness without thinking, but tweets can arguably show what they really feel in that moment. However, it should also be acknowledged that tweets are short and very often have little context. They can also be sent in the heat of the moment and so may not always portray the more nuanced beliefs and perceptions of an individual tweeter. An organisation’s tweets tend to be more structured and strategised, but they may not always be shared by those within the organisation.

## 5. Conclusions

Analysing tweets around COVID-19 and the NHS during the first lockdown in the UK highlights the importance of bearing witness to the experiences of not only NHS staff but the general population as everyone tried to make sense of the impact of a global pandemic. Looking back from the perspective of the third lockdown, some of these themes re-emerge more strongly in relation to COVID-19 denial and in particular the resistance of the ‘COVID-19 deniers’ in appreciating the traumatic impact on NHS staff of caring for the surge of sick and dying patients and their grieving families. This shift in sentiment was captured in a tweet by a high-profile medical academic on 7 February 2021 ‘Wave 2/3 abuse instead of clapping’ which also linked to the newspaper article by frontline medic Dr Rachel Clarke entitled ‘I’ve been called Satan’ [28], where she describes abuse being directed to her from ‘COVID-19 deniers’. While it is beyond the scope of this paper to systematically examine experiences of the NHS from the perspective of subsequent COVID-19 lockdowns in the UK, it is nonetheless already clear that there have been notable shifts with regard to sentiment directed towards NHS staff and the NHS overall. While hero worship of staff and staff betrayal from lack of PPE were prominent themes in the first lockdown, later lockdowns are characterised by different themes, including abuse directed towards staff, COVID-19 denial and a surge in patient demand overwhelming hospital capacity. It will be important to understand these narratives further as they are likely to shape NHS staff experience of working in the pandemic, their relationship to the NHS as their employer, and the ongoing psychological impact on the NHS workforce [31]. Further research exploring social media narratives around COVID-19 and the NHS should help in working towards devising suitable tailored support for staff in the future and acknowledging the profound impact that the pandemic has had.

## Figures and Tables

**Table 1:** Tweets per week by hashtag.

Week Starting	BOOBORIS	BOOFORBORIS	CLAPFOROURCARERS	CLAPFOROURNHS	NHS COIVD19UK	NHS COVID_19	NHS COVID19	NHS COVID19IRELAND	NHS PPE
30-March	9	148		13,355			5094	2	
6-April	7	211		7235		443	7257	3	
13-April	3	47		12,457	296	2013	7222	6	
20-April	1	24		9027	601	1020	4761	2	
27-April	7	23	1144	1089	632	636	4929	4	
4-May	25	37	4671		395	486	3565	1	
11-May	3	34	3417		458	267	2714	2	
18-May	51	3621	2753		424	668	1766		177
25-May	95	5576	2302		289	222	1546		374
1-June	4	135	446		179	135	1357		325
8-June	1	49	140		123	88	1155	2	224
15-June	1	14	62		199	160	1127		215
22-June		11	122		164	99	1060		154
29-June		4	18		49	36	341		34
**Grand Total**	**207**	**9934**	**15,075**	**43,163**	**3809**	**6273**	**43,894**	**22**	**1503**
